# Influencing factors of oral frailty in Chinese maintenance hemodialysis patients: a Bayesian network analysis

**DOI:** 10.1080/0886022X.2026.2673605

**Published:** 2026-06-02

**Authors:** Yiqian Fang, Qian Jiang, Rong Zeng, Xueqin Gan, Ping Wei, Juan Du, Xiaoxia Lin, Qian Yang

**Affiliations:** a Chengdu Medical College, Chengdu, China; b The Peoples Hospital of Jianyang City, Jianyang, China; c The Second Affiliated Hospital of Chengdu Medical College, Chengdu, China; d The First Affiliated Hospital of Chengdu Medical College, Chengdu, China

**Keywords:** Hemodialysis, oral frailty, Health Ecology Model (HEM), Bayesian, risk factors

## Abstract

This multicenter cross-sectional study analyzed the prevalence and multidimensional determinants of oral frailty in maintenance hemodialysis (MHD) patients using the Health Ecology Model (HEM) and provided evidence for targeted clinical interventions. Patients undergoing MHD across four tertiary hospitals in China were included (*n* = 680). Independent influencing factors were identified *via* binary logistic regression, and a Bayesian network model was constructed using R 4.3.3 and GeNIe 4.0 for risk inference. The prevalence of oral frailty was 42.94%. Binary logistic regression identified 11 independent influencers. Poor oral health (OR = 5.288), polypharmacy (OR = 5.194), general frailty (OR = 4.465), depression (OR = 3.393), malnutrition (OR = 3.093), eating alone (OR = 2.310), age ≥60 years (OR = 2.221), and meat-dominant dietary pattern (OR = 2.061) were risk factors, whereas higher education (ORs: 0.139–0.453), higher monthly income (ORs: 0.154–0.524), and better social support (ORs: 0.036–0.203) were protective factors (all *p* < 0.05). Furthermore, the Bayesian network model demonstrated that polypharmacy, malnutrition, eating alone, and general frailty directly influenced oral frailty, whereas sociodemographic factors, dietary pattern, poor oral health, depression, and social support exerted indirect effects. These results suggest that early risk identification and multilevel interventions should be incorporated into routine dialysis care to mitigate the risk of oral frailty.

## PLAIN LANGUAGE SUMMARY

In this study of 680 patients receiving long-term hemodialysis, a significant portion (42.94%) experienced oral frailty, a condition characterized by a decline in mouth functions such as chewing and swallowing. These oral functional declines were closely linked to a complex web of direct factors—such as taking multiple medications, poor nutrition, eating alone, and overall physical weakness—as well as indirect factors like income, education, and mental health. Implementing comprehensive, multilevel care strategies (e.g., providing nutritional support, optimizing medications, and enhancing social support) can help identify at-risk individuals early, suggesting a practical target for preventing further oral and general health deterioration in dialysis care.

## 1. Introduction

Chronic kidney disease (CKD) has emerged as a major global public health concern, affecting more than 850 million individuals worldwide [1]. China bears the highest global burden of CKD, with an estimated 132.3 million affected individuals. As the disease progresses, CKD inevitably advances to end-stage renal disease (ESRD), characterized by a complete loss of physiological renal function and the subsequent need for renal replacement therapy. Maintenance hemodialysis (MHD) serves as the primary modality of renal replacement therapy for patients with ESRD [2]. Driven by continuous advances in medical technology and the progressive expansion of national health insurance policies, the MHD patient population has surged rapidly [3]. According to statistics from the Chinese National Renal Data System (CNRDS; https://www.cnrds.net), as of the end of 2024, a total of 1.027 million patients with end-stage kidney disease (ESKD) in mainland China relied on life-sustaining hemodialysis.

Over the course of prolonged MHD treatment, patients inevitably suffer adverse oral health effects stemming from disease-induced pathophysiological changes and treatment-related tissue damage [4]. Consequently, these patients frequently encounter issues such as gingival swelling, oral mucosal lesions, periodontitis, and an elevated risk of infection [5–7], all of which contribute to an increased susceptibility to oral frailty [8]. Oral frailty (OF) is defined as a mild, age-related decline in oral function accompanied by diminished physical and psychological capacities, representing a reversible early stage of overall frailty [9,10]. Epidemiological data indicate that the prevalence of oral frailty is approximately 20.4% among community-dwelling older adults in Japan [11] and 33.8% among those in China [12]. However, the prevalence surges to 41.2% among MHD patients [13], underscoring a significantly more pronounced burden of oral frailty in this clinical population compared to the general public. Oral frailty not only compromises fundamental physiological functions, such as mastication and swallowing—thereby exacerbating nutritional risks, physical frailty, and mortality rates [14]—but also negatively impacts patients’ psychological well-being and social participation. These physiological and psychosocial detriments intricately intertwine, ultimately devastating the patients’ overall quality of life [5,15]. Given the strong association between oral frailty and adverse health outcomes, along with its potential reversibility, early identification of its influencing factors is essential.

Current studies on oral frailty in patients undergoing MHD predominantly rely on conventional statistical methods [14,16], which are limited in their ability to capture complex interactions among variables. Bayesian network analysis, capable of constructing directed acyclic graphs and conditional probability tables, can intuitively reflect dependencies among variables [17] and supports probabilistic inference and causal exploration [18]. The Health Ecology Model (HEM) emphasizes that health outcomes result from the systematic interaction of multilevel factors [19], thereby providing an ideal theoretical framework for systematically interpreting the multidimensional determinants of oral frailty in MHD patients.

Grounded in the HEM, this study aims to construct a bayesian network model for oral frailty in MHD patients. We systematically investigate the influencing factors and their intrinsic associations across five dimensions: personal traits, psychological and behavioral characteristics, interpersonal networks, living and working conditions, and the policy environment. Ultimately, this approach facilitates risk inference and prediction of oral frailty, providing an evidence-based reference for the early clinical identification of at-risk patients and the formulation of targeted interventions.

## 2. Methods

### 2.1. Study design and participants

This was a multicenter, cross-sectional study. Between November 2024 and May 2025, a convenience sampling method was employed to recruit patients undergoing MHD from the blood purification centers of four tertiary Grade A hospitals in Chengdu, China. The inclusion criteria were as follows: (1) age ≥18 years; (2) diagnosis of ESRD meeting the criteria outlined in the 2024 Kidney Disease Outcomes Quality Initiative (KDOQI) guidelines [20], accompanied by regular hemodialysis treatment for ≥3 months; (3) provision of voluntary, written informed consent to participate in the study. The exclusion criteria were as follows: (1) presence of audiovisual, speech, psychiatric, or cognitive impairments; (2) presence of severe infections or concurrent systemic diseases (e.g., malignant tumors, autoimmune diseases); (3) a history of oral trauma or severe oral diseases within the previous three months, or a confirmed diagnosis of organic salivary gland diseases or Sjögren’s syndrome.

The sample size was calculated using the formula for the overall rate in observational studies: ${n=[u}_{1-\alpha /2}^{2}\pi (1-\pi )]/\delta^{2}$. Based on previous literature [13], the prevalence of oral frailty among MHD patients was estimated at 41.2%. With a 95% confidence level, a 5% margin of error, and an anticipated non-response rate of 20%, the minimum required sample size was calculated to be 467. Among the 715 initially recruited participants, 10 patients refused to cooperate after signing the informed consent, and 25 had incomplete laboratory data. Given the small proportion of missing data (4.9%), we adopted a complete-case analysis approach and excluded these 35 participants, resulting in a final sample of 680 valid cases. The study protocol was approved by the Ethics Committee of Chengdu Medical College (Approval No.: 2024NO.69) and was conducted in strict accordance with the principles of the Declaration of Helsinki. Written informed consent was obtained from all participants.

### 2.2. Variable selection

**Figure 1.**
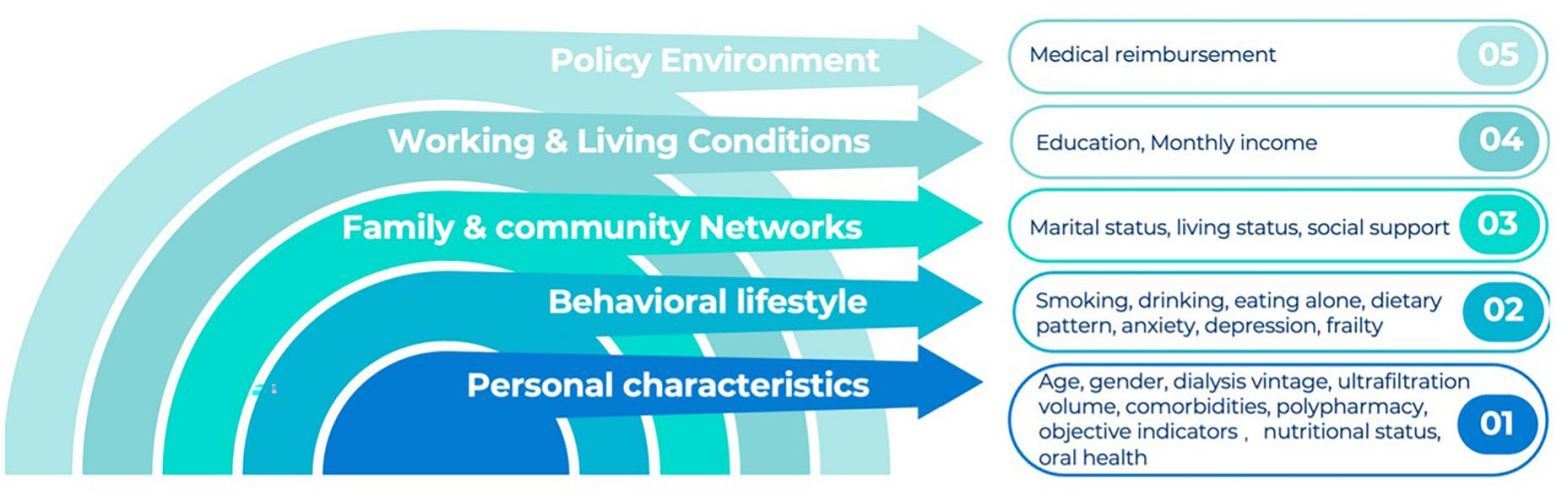
Analytical framework of influencing factors for oral frailty in MHD patients based on the HEM.

Based on the five domains of the HEM, potential factors that may influence oral frailty in MHD patients were identified and categorized through a literature review, expert consultation, and group discussions as follows: (1) Personal characteristics: age, sex, dialysis vintage, ultrafiltration volume, comorbidities, polypharmacy, body mass index (BMI), grip strength, nutritional status, oral health status, biochemical indicators; (2) Psychological and behavioral lifestyle: smoking, drinking, eating alone, dietary pattern, general frailty, anxiety, depression; (3) Family and community networks: marital status, living status, social support; (4) Working and living conditions: education, monthly income; (5) Policy environment: medical reimbursement (Figure 1).

### 2.3. Research instruments

#### 2.3.1. General information questionnaire

A self-designed questionnaire was developed by the researchers based on a literature review to collect sociodemographic and disease-related data. (1) Sociodemographic information: age, sex, marital status, education, living status, monthly income, medical reimbursement, smoking, drinking, eating alone, and dietary pattern; (2) Clinical information: dialysis vintage, ultrafiltration volume, and polypharmacy (≥5 drugs); (3) Objective indicators: body mass index (BMI), handgrip strength, hemoglobin, ferritin, high-sensitivity C-reactive protein (hs-CRP), serum albumin, serum creatinine, blood urea nitrogen (BUN), serum potassium, serum calcium, serum phosphorus, intact parathyroid hormone (iPTH), triglycerides, total cholesterol, and dialysis adequacy (Kt/V).

#### 2.3.2. Oral frailty index-8 (OFI-8)

Developed by Tanaka et al. [21], this index is used to evaluate oral frailty. It comprises 8 items covering denture use (1 item), swallowing ability (1 item), social participation (1 item), oral health-related behaviors (2 items), and chewing ability (3 items). The total score ranges from 0 to 11, with a score ≥4 indicating the presence of oral frailty [22]. In this study, this cutoff was likewise used as the threshold for oral frailty. The original scale had a Cronbach’s α coefficient of 0.693, while in this study it was 0.727, indicating good internal consistency reliability.

#### 2.3.3. Oral health assessment tool (OHAT)

Revised by Chalmers et al. [23] based on the Brief Oral Health Status Examination (BOHSE), this tool evaluates patients’ oral health status. The tool consists of 8 items assessing the lips, tongue, gums and tissues, saliva, natural teeth, dentures, oral cleanliness, and dental pain. Each item is scored on a 3-point scale, yielding a total score ranging from 0 to 16. A score ≥3 indicates poor oral health, whereas a score < 3 indicates good oral health [24]. Higher scores reflect worse oral health status. The tool features concise items and easy administration, making it suitable for non-dental healthcare professionals. The original scale had a Cronbach’s α coefficient of 0.780, while in this study it was 0.915, indicating good reliability.

#### 2.3.4. Modified quantitative subjective global assessment (MQSGA)

Developed by Kalantar-Zadeh et al. [25], this scale evaluates the nutritional status of MHD patients. It includes 7 components: weight change, dietary intake, gastrointestinal symptoms, functional capacity, comorbidities, and depletion of subcutaneous fat and muscle mass. Each component is assigned a score from 1 to 5, resulting in a total score ranging from 7 to 35. A total score ≤10 indicates normal nutritional status, while a score of 11–35 indicates malnutrition [26]. Higher scores signify poorer nutritional status.

#### 2.3.5. Age-adjusted Charlson comorbidity index (ACCI)

The ACCI [27] was used to evaluate the comorbidity status of MHD patients. Based on the original Charlson Comorbidity Index, this scale adjusts the scoring criteria for age: 1 point is added for every decade over 40 years of age (i.e., 1 point for 50–59 years, 2 points for 60–69 years, 3 points for 70–79 years, and 4 points for ≥ 80 years). The scale comprises 19 comorbidity items divided into four subsets (ten 1-point items, six 2-point items, one 3-point item, and two 6-point items). Because all participants in this study were CKD patients, the item for moderate-to-severe CKD was removed. The total score ranges from 0 to 35, with higher scores indicating a heavier comorbidity burden.

#### 2.3.6. Grip strength (HGS)

Handgrip strength was measured using an EH108 electronic hand dynamometer (Guangdong Xiangshan Weighing Apparatus Group, China). Patients were instructed to stand naturally and grip the dynamometer using their dominant hand or the hand without an arteriovenous fistula. The measurement was performed twice, and the maximum reading was recorded.

#### 2.3.7. Hospital anxiety and depression scale (HADS)

Developed by Zigmond and Snaith [28], this scale is primarily used to screen for anxiety and depression among patients in general hospitals. It consists of 14 items divided into two subscales: anxiety (HADS-A) and depression (HADS-D), each containing 7 items. The score for each subscale ranges from 0 to 21. A score of 0–7 is classified as normal, and higher scores indicate more severe levels of anxiety or depression. The Cronbach’s α coefficient of the scale was 0.866.

#### 2.3.8. Perceived social support scale (PSSS)

Developed by Zimet et al. [29], this scale assesses the level of social support perceived by the subjects. It consists of 12 items encompassing three dimensions: family support, friend support, and other support. Each item is scored on a 7-point scale, resulting in a total score ranging from 12 to 84. Higher scores indicate a higher level of perceived overall social support. Total scores of 12–36, 37–60, and 61–84 represent low, moderate, and high levels of support, respectively. The Cronbach’s α coefficient of the scale was 0.821.

#### 2.3.9. Groningen frailty indicator (GFI)

GFI was utilized to evaluate the frailty status of the patients. Originally developed by Steverink [30], the scale encompasses four dimensions: physical, cognitive, psychological, and social. Each item is scored as either 0 or 1, yielding a total score ranging from 0 to 15. A total score > 4 indicates the presence of frailty, with higher scores reflecting a greater severity of frailty. In the Chinese population, the scale has demonstrated a Cronbach’s α coefficient of 0.712 and a test-retest reliability coefficient of 0.939.

### 2.4. Statistical analysis

Statistical analyses were performed using SPSS version 27.0 and R version 4.3.3 software. Normally distributed continuous variables are presented as mean ± standard deviation (SD), while non‑normally distributed continuous variables are presented as median (*P*_25_, *P*_75_), categorical variables are presented as frequencies (%). In univariate analysis, independent samples *t*-test, Mann-Whitney *U* test, or chi-square test was used as appropriate for between-group comparisons. Taking the occurrence of oral frailty as the dependent variable, variables that were statistically significant in the univariate analysis were entered as independent variables into a binary logistic regression model for multivariate analysis. Binary logistic regression analysis was employed to determine the variable nodes for the Bayesian network, and the “bnlearn” package was used to construct the model. Structure learning was performed using the Max-Min Hill-Climbing (MMHC) algorithm. The initial network was then refined by consulting relevant literature and expert opinion, which involved removing unreasonable directed edges and adding necessary ones. Parameter learning was subsequently conducted using maximum likelihood estimation to obtain the conditional probability tables for each node. Finally, GeNIe 4.0 software was used to visualize the Bayesian network structure, perform sensitivity analysis, and generate conditional probability tables. Model performance was evaluated using the Receiver Operating Characteristic (ROC) curve. The significance level was set at *α* = 0.05 (two-tailed), and *p* < 0.05 was considered statistically significant.

## 3. Result

### 3.1. Characteristics of participants

A total of 680 MHD patients were included in this study. Among them, 401 (59.0%) were male and 279 (41.0%) were female, and 292 patients presented with oral frailty. The results of the univariate analysis revealed statistically significant differences between MHD patients with and without oral frailty in terms of age, polypharmacy, handgrip strength, oral health status, nutritional status, eating alone, dietary pattern, general frailty, depression, social support, education, and monthly income (Table 1).

**Table 1.** Characteristics of MHD patients according to oral frailty status.

Variable	Non-oral frailty (*n* = 388)	oral frailty (*n* = 292)	*t*/*z*/*χ^2^*	*p*
	*n*(%)or MSD(MinMax)	*n*(%)or MSD(MinMax)		
**Personal characteristics**				
Age (years)			49.341	<0.001
<60	229 (59.02)	93 (31.85)		
≥60	159 (40.98)	199 (68.15)		
Sex			0.836	0.360
Male	223 (57.47)	178 (60.96)		
Female	165 (42.53)	114 (39.04)		
Dialysis vintage (months)	48.00 (24.00, 96.00)	36.00 (20.00, 85.00)	−1.601	0.109
Ultrafiltration volume (L)	2.23 ± 0.81	2.12 ± 0.85	1.800	0.072
ACCI	3.00 (2.00, 4.00)	3.00 (1.00, 6.00)	−1.751	0.080
Polypharmacy(≥5drugs)			92.887	<0.001
No	224 (57.73)	61 (20.89)		
Yes	164 (42.27)	231 (79.11)		
BMI (kg/m^2^)			0.115	0.990
Underweight (<18.50)	58 (14.95)	43 (14.73)		
Normal weight (18.50–23.99)	194 (50.00)	146 (50.00)		
Overweight (24.00–27.99)	99 (25.52)	73 (25.00)		
Obes (≥28.00)	37 (9.54)	30 (10.27)		
HGS (kg)	22.00 (18.00, 25.00)	18.00 (16.00, 20.00)	−9.526	<0.001
Objective indicators				
Hemoglobin (g/L)	112.07 ± 19.71	111.57 ± 18.56	0.338	0.735
Ferritin ( μ g/L)	103.75 (51.32, 243.30)	95.05 (47.28, 217.95)	−0.872	0.383
hs-CRP (mg/L)	3.72 (1.19, 9.68)	4.40 (1.44, 14.08)	−1.925	0.054
Serum albumin (g/L)	38.50 (29.50, 40.30)	37.65 (33.30, 40.13)	−0.986	0.324
Serum creatinine ( μ mol/L)	852.50 (623.50, 1027.00)	837.50 (612.50, 1012.50)	−0.519	0.603
BUN (mmol/L)	22.85 (16.26, 27.47)	23.28 (17.94, 28.07)	−1.576	0.115
Serum potassium (mmol/L)	4.80 ± 0.82	4.79 ± 0.85	0.195	0.845
Serum calcium (mmol/L)	2.20 (2.08, 2.34)	2.22 (2.09, 2.36)	−0.954	0.340
Serum phosphorus (mmol/L)	1.86 (1.41, 2.26)	1.86 (1.40, 2.34)	−0.241	0.809
iPTH (pg/mL)	294.30 (174.30, 513.30)	309.40 (176.75, 526.30)	−0.553	0.580
Triglycerides (mmol/L)	1.55 (1.40, 1.80)	1.60 (1.40, 1.80)	−1.529	0.126
Total cholesterol (mmol/L)	4.25 ± 0.89	4.36 ± 0.91	−1.610	0.108
Kt/V	1.39 ± 0.35	1.36 ± 0.37	0.906	0.365
Poor oral health			20.791	<0.001
No	120 (30.93)	46 (15.75)		
Yes	268 (69.07)	246 (84.25)		
Malnutrition			106.701	<0.001
No	235 (60.57)	61 (20.89)		
Yes	153 (39.43)	231 (79.11)		
**Psychological and behavioral lifestyle**				
Smoking			0.203	0.652
No	300 (77.32)	230 (78.77)		
Yes	88 (22.68)	62 (21.23)		
Drinking			0.100	0.752
No	299 (77.06)	222 (76.03)		
Yes	89 (22.94)	70 (23.97)		
Eating alone			26.378	<0.001
No	222 (57.22)	109 (37.33)		
Yes	166 (42.78)	183 (62.67)		
Dietary pattern			15.692	<0.001
Balanced	226 (58.25)	136 (46.58)		
Meat-dominant	81 (20.88)	100 (34.25)		
Vegetable-dominant	81 (20.88)	56 (19.18)		
General frailty			45.276	<0.001
No	109 (28.09)	22 (7.53)		
Yes	279 (71.91)	270 (92.47)		
Depression			13.294	<0.001
No	314 (80.93)	187 (64.04)		
Yes	74 (19.07)	105 (35.96)		
Anxiety			1.883	0.170
No	273 (70.36)	191 (65.41)		
Yes	115 (29.64)	101 (34.59)		
**Family and community networks**				
Marital status			4.023	0.134
Single/Unmarried	22 (5.67)	14 (4.79)		
Married	327 (84.28)	234 (80.14)		
Divorced/Widowed	39 (10.05)	44 (15.07)		
Living status			0.032	0.857
Not alone	338 (87.11)	253 (86.64)		
Alone	50 (12.89)	39 (13.36)		
Social support			136.615	<0.001
Low	3 (0.77)	38 (13.01)		
Moderate	93 (23.97)	159 (54.45)		
High	292 (75.26)	95 (32.53)		
**Working and living conditions**				
Education			84.934	<0.001
Primary school or below	73 (18.81)	123 (42.12)		
Secondary school	215 (55.41)	160 (54.79)		
College or above	100 (25.77)	9 (3.08)		
Monthly income (CNY)			158.602	<0.001
<1000	59 (15.21)	145 (49.66)		
1000–3000	96 (24.74)	104 (35.62)		
3001–5000	129 (33.25)	30 (10.27)		
>5000	104 (26.80)	13 (4.45)		
**Policy environment**				
Medical reimbursement			3.626	0.305
Urban employee	189 (48.71)	135 (46.23)		
Urban resident	75 (19.33)	48 (16.44)		
NCMS	111 (28.61)	102 (34.93)		
Out-of-pocket	13 (3.35)	7 (2.40)		

*Note:* ACCI: age-adjusted Charlson comorbidity index; BMI: body mass index; HGS: Grip strength; hs-CRP: high-sensitivity C-reactive protein; BUN: blood urea nitrogen; NCMS: New Cooperative Medical Scheme: CNY: Chinese Yuan.

### 3.2. Multifactorial analysis

A binary logistic regression analysis was conducted with the presence of oral frailty as the dependent variable and the statistically significant variables identified in the univariate analysis as the independent variables. Multicollinearity diagnostics showed that the tolerance of all independent variables included in the regression equation was greater than 0.1, and the variance inflation factor (VIF) was less than 5, indicating that there was no significant multicollinearity among the independent variables. The multivariable analysis demonstrated that age, education, monthly income, eating alone, dietary pattern, polypharmacy, malnutrition, general frailty, depression, and social support were independent influencing factors for oral frailty in MHD patients (Table 2).

**Table 2.** Binary logistic regression analysis of influencing factors for oral frailty in patients undergoing MHD.

Variable	Reference	B	SE	Wald	OR (95%CI)	*p*
Age (years)	<60	0.798	0.260	9.414	2.221 (1.334, 3.699)	0.002
Education	Primary school or below					
Secondary school		−0.792	0.266	8.830	0.453 (0.269, 0.764)	0.003
College or above		−1.975	0.544	13.177	0.139 (0.048, 0.403)	<0.001
Monthly income (CNY)	<1000					
1000–3000		−0.645	0.287	5.049	0.524 (0.299, 0.921)	0.025
3001–5000		−1.864	0.389	22.986	0.155 (0.072, 0.332)	<0.001
>5000		−1.873	0.476	15.483	0.154 (0.060, 0.391)	<0.001
HGS (kg)		−0.019	0.032	0.352	0.981 (0.922, 1.044)	0.553
Eating alone	No	0.837	0.249	11.338	2.310 (1.419, 3.761)	0.001
Dietary pattern	Balanced					
Meat-dominant		0.723	0.318	5.168	2.061 (1.105, 3.845)	0.023
Vegetable-dominant		0.520	0.327	2.532	1.683 (0.886, 3.194)	0.112
Polypharmacy	No	1.647	0.259	40.312	5.194 (3.123, 8.637)	<0.001
Malnutrition	No	1.129	0.273	17.050	3.093 (1.810, 5.286)	<0.001
General frailty	No	1.496	0.353	17.985	4.465 (2.236, 8.916)	<0.001
Poor oral health	No	1.665	0.304	30.018	5.288 (2.914, 9.594)	<0.001
Depression	No	1.222	0.293	17.395	3.393 (1.911, 6.024)	<0.001
Social support	Low					
Moderate		−1.596	0.791	4.069	0.203 (0.043, 0.956)	0.044
High		−3.338	0.787	17.976	0.036 (0.008, 0.166)	<0.001

*Note:* SE: standard error; OR: odds ratio; CI: confidence interval.

### 3.3. Construction of the Bayesian network model

**Figure 2.**
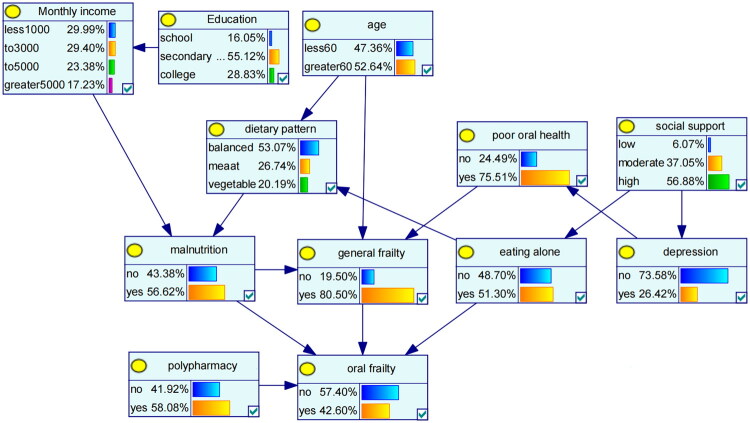
Bayesian network for oral frailty in MHD patients.

The 11 variables that demonstrated statistical significance in the multivariable logistic regression analysis were selected as network nodes. Subsequently, a Bayesian network model, comprising 12 nodes and 15 directed edges, was constructed to delineate the associated factors for oral frailty in MHD patients. The results indicated that polypharmacy, malnutrition, eating alone, and general frailty directly influenced the occurrence of oral frailty. Specifically, both malnutrition and general frailty were directly associated with oral frailty; additionally, malnutrition exerted an indirect effect on oral frailty *via* general frailty. Eating alone directly influenced oral frailty, while social support indirectly affected oral frailty *via* eating alone. Furthermore, eating alone was indirectly associated with oral frailty *via* dietary pattern and malnutrition. Age exerted an indirect effect on oral frailty *via* general frailty, as well as *via* dietary pattern and malnutrition. Education was indirectly linked to oral frailty *via* monthly income and malnutrition. Lastly, depression was indirectly associated with oral frailty *via* oral health status and general frailty (Figure 2).

### 3.4. Inference of the Bayesian network model

**Figure 3.**
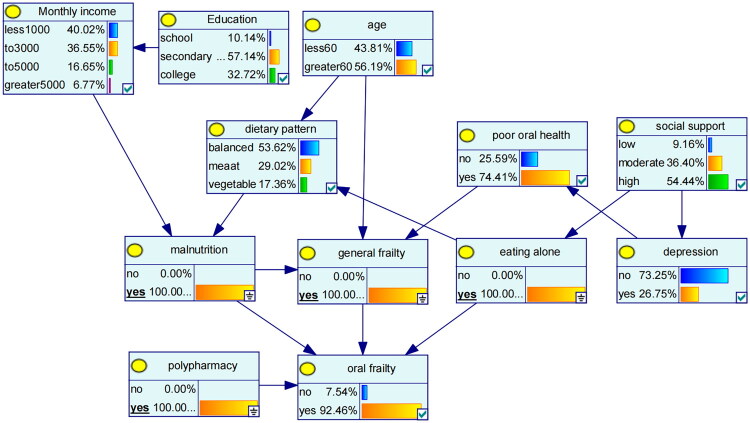
Risk inference of oral frailty in MHD patients based on Bayesian network.

Table 3 presents the conditional probabilities for the oral frailty node, illustrating the probabilistic dependencies among polypharmacy, eating alone, malnutrition, and general frailty. The numerical values within the nodes in Figure 3 indicate the levels of the corresponding variables and their occurrence probabilities in the training set, intuitively depicting the probabilistic dependencies among the variables. Each node has two levels (‘yes’ and ‘no’). When the four factors—polypharmacy, malnutrition, eating alone, and general frailty—are all present (i.e., each has a probability of 100%), the probability of oral frailty being ‘yes’ reaches its highest level (92.46%).

**Table 3.** Probability of oral frailty conditions in MHD patients.

Polypharmacy	Malnutrition	Eating alone	General frailty	oral frailty	
				No	Yes
No	No	No	No	91.67%	8.33%
No	No	No	Yes	82.50%	17.50%
No	No	Yes	No	92.11%	7.89%
No	No	Yes	Yes	89.34%	10.66%
No	Yes	No	No	79.17%	20.83%
No	Yes	No	Yes	66.98%	33.02%
No	Yes	Yes	No	91.18%	8.82%
No	Yes	Yes	Yes	55.66%	44.34%
Yes	No	No	No	92.11%	7.89%
Yes	No	No	Yes	72.50%	27.50%
Yes	No	Yes	No	86.11%	13.89%
Yes	No	Yes	Yes	50.00%	50.00%
Yes	Yes	No	No	79.55%	20.45%
Yes	Yes	No	Yes	38.42%	61.58%
Yes	Yes	Yes	No	25.00%	75.00%
Yes	Yes	Yes	Yes	7.54%	92.46%

### 3.5. The Bayesian network model validation and sensitivity analysis

**Figure 4.**
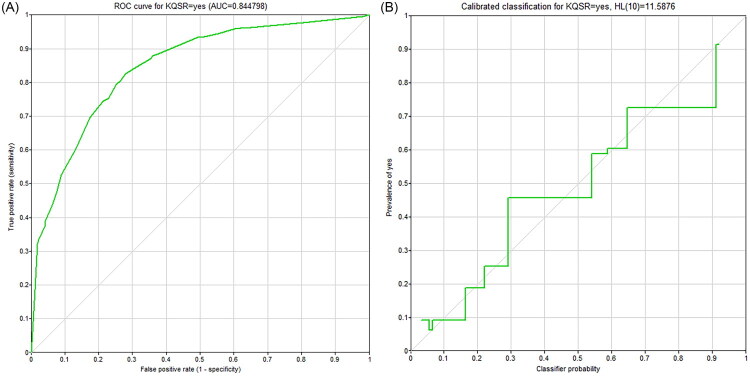
The Bayesian network model validation (A) ROC Curve of Bayesian Network Prediction Model; (B) Calibration Curve of Bayesian Network Prediction Model.

**Figure 5.**
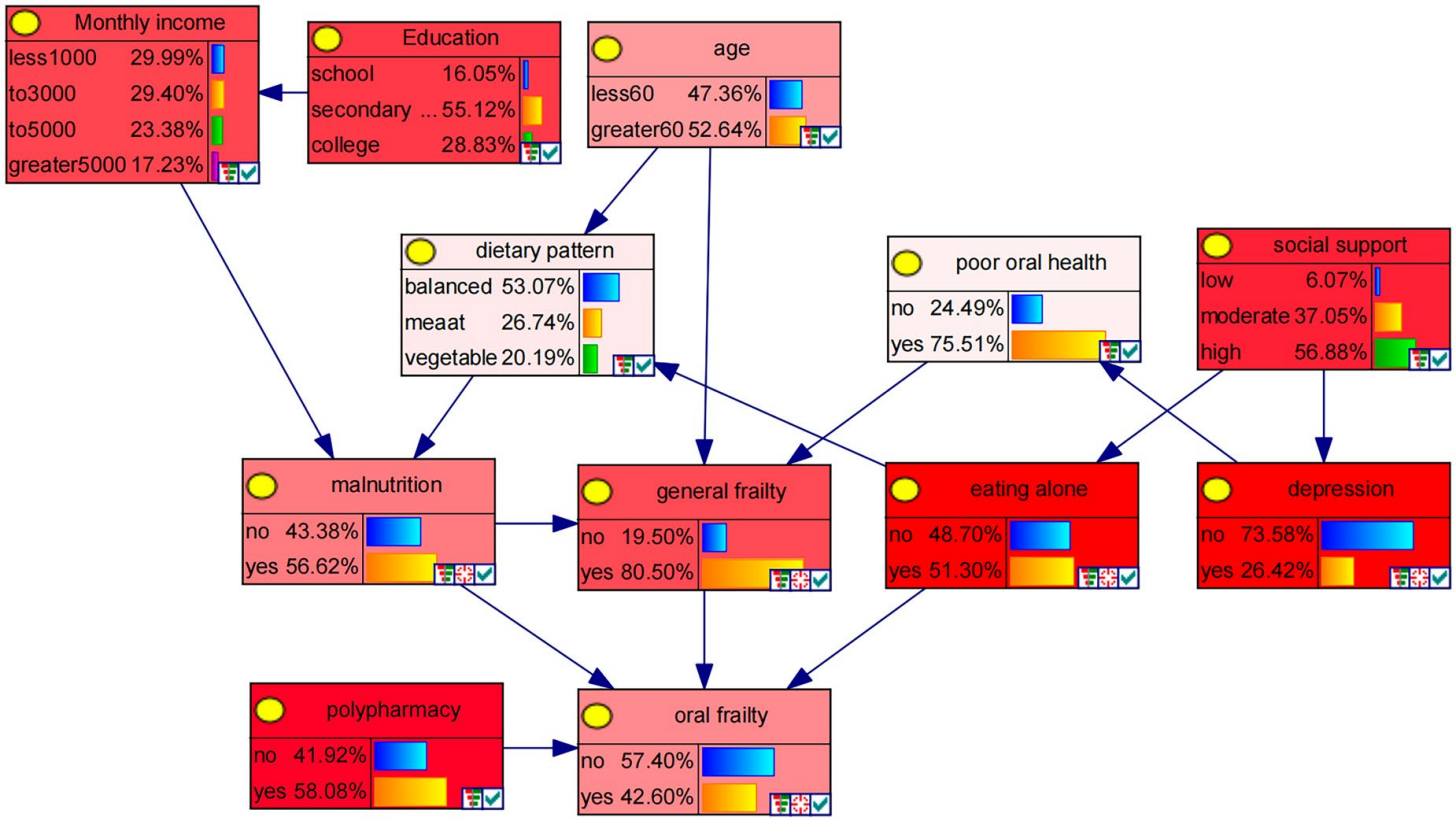
Sensitivity analysis of the oral frailty node.

The area under the receiver operating characteristic (ROC) curve (AUC) was 0.845, with a specificity of 0.814 and a sensitivity of 0.843, indicating good discriminative ability of the model (Figure 4A). In terms of model calibration, the calibration curve showed good agreement between the predicted probabilities and the observed frequencies (Figure 4B). The Hosmer‑Lemeshow test yielded a chi‑square statistic of 11.588 (df = 8, *p* = 0.170), indicating no evidence of significant miscalibration. With oral frailty set as the target node, sensitivity analysis results are shown in Figure 5 and Table 4.

**Table 4.** Sensitivity of various indexes in prediction of angina pectoris.

Node	Sensitivity index
Eating alone	0.569
Depression	0.569
Polypharmacy	0.301
Social support	0.278
Education	0.249
Monthly income	0.235
General frailty	0.227
Malnutrition	0.159
Age	0.117

## 4. Discussion

### 4.1. Prevalence of oral frailty in MHD patients

Based on the HEM framework, this study constructed a Bayesian network model for oral frailty in MHD patients and explored the intrinsic relationships among various predictors. The results indicated that polypharmacy, malnutrition, eating alone, and general frailty were directly associated with oral frailty. Meanwhile, age, education, monthly income, dietary pattern, depression, poor oral health, and social support exerted indirect effects on oral frailty. This indicates that the oral health outcomes of MHD patients are the product of the synergistic effects of multilevel determinants. These outcomes are primarily driven by interpersonal networks, working and living conditions (midstream factors), and behavioral characteristics (downstream factors), whereas the direct impact of the policy environment (upstream factors) is limited, which aligns with the core tenets of the HEM.

The prevalence of oral frailty among the MHD patients investigated in this study was 42.94%, which is higher than that reported in community-dwelling older adults (20.7%–33.8%) [12,31], suggesting that this population faces a severe risk of accelerated oral function decline. This finding is consistent with several recent surveys targeting Chinese MHD patients. For instance, Dou et al. [13] reported a prevalence of 41.2%, and Chen et al. [32] reported a prevalence of 45.2% among older MHD patients, indicating the universality of oral frailty in this population. A plausible explanation is that MHD patients are more susceptible to a series of oral health issues—such as periodontal disease, reduced salivary secretion, uremic fetor, and dysphagia—due to renal replacement therapy, pharmacological treatments, fluid restriction, and immunosuppression [33], which significantly elevate the risk of oral frailty. However, in actual clinical practice, the coverage rate of oral health education for MHD patients is less than 3% [34]. Therefore, clinical management must prioritize the oral healthcare of MHD patients by implementing early identification and comprehensive interventions to break the vicious cycle of oral frailty and systemic health deterioration.

### 4.2. Analysis of influencing factors for oral frailty in MHD patients

#### 4.2.1. Personal characteristics

The results of this study show that within the personal characteristics layer, advanced age, polypharmacy, poor oral health, and malnutrition are risk factors for oral frailty in MHD patients. Age is a key indicator of physiological aging; the older the MHD patient, the higher their risk of oral frailty. With increasing age, systemic physiological reserve declines and regenerative capacity diminishes. This gradually leads to issues such as decreased alkaline phosphatase activity in periodontal ligament cells, reduced regenerative and osteogenic activity of periodontal ligament stem cells, physiological gingival recession, and cementum demineralization and softening, contributing to diseases like periodontitis and dental caries, and ultimately resulting in oral frailty. Secondly, polypharmacy, a common clinical feature among MHD patients, has a direct impact on oral frailty, consistent with the findings of Chen et al. [16]. Side effects of multiple medications (e.g., non-steroidal anti-Inflammatory drugs, phosphate binders, anticholinergics) can directly cause oral complications such as angular cheilitis and reduced saliva secretion [35], impairing oral self-cleaning ability and increasing the risk of mucosal lesions, dental caries, and *Candida* infections. Poor oral health is a risk factor for oral frailty, primarily manifesting as reduced saliva flow, dental wear or caries, and poor oral hygiene (plaque, calculus, or halitosis). Saliva is rich in glycoproteins, antimicrobial proteins, and defensins, which inhibit the adhesion of oral fungi to epithelial cells. Its reduction facilitates oral fungal colonization [36], disrupting the oral microecological balance and leading to oral frailty. Furthermore, reduced tooth number or pain directly impairs chewing mechanical efficiency, while poor oral hygiene and gum issues collectively compromise mucosal defense [37]. Dysphagia affects swallowing ability, further exacerbating the deterioration of oral frailty. Metabolic disturbances caused by uremic toxins and dialysis-related factors predispose MHD patients to impaired nutrient absorption and loss, significantly increasing the risk of malnutrition. The study results show that malnutrition is an independent influencing factor for oral frailty and also has a direct impact on it, aligning with the findings of Dou et al. [13]. Malnutrition leads to sarcopenia, reduced bone mass, and decreased physical function, which in turn cause a decline in oral function. The decline in oral function then forces patients to narrow their food choices, avoiding nutrient-dense foods that require adequate mastication. This restriction in eating behavior further worsens nutritional status, creating a self-reinforcing negative cycle [38]. Therefore, focusing on oral health, conducting regular oral examinations to promptly identify and address issues despite the uncontrollable factor of age, prioritizing simplified medication regimens to ensure safe, accurate, and rational drug use, and emphasizing nutritional screening and support will help reduce the oral health burden in MHD patients.

#### 4.2.2. Psychological and behavioral lifestyle

The psychological and behavioral lifestyle layer is the intermediate level of the HEM model. This study indicates that eating alone, dietary pattern, general frailty, and depression are influencing factors for the occurrence of oral frailty in MHD patients. Research shows that MHD patients who eat alone have a higher risk of oral frailty, which aligns with the findings of Tanaka et al. [21]. This may be because reduced conversation and chewing during solitary meals contribute to a decline in oral function. Regarding dietary pattern, this study also found that compared to MHD patients with a balanced diet of meat and vegetables, those with a predominantly meat-based diet had a higher risk of developing oral frailty. This result is consistent with the report by Hoshino et al. [39]. Dietary restrictions between dialysis sessions may be accompanied by insufficient intake of fatty acids, vitamins C and E, β-carotene, fiber, calcium, dairy products, fruits, and vegetables, increasing the risk of periodontal disease [40]. This makes teeth more prone to loss, directly increasing the oral burden. Simultaneously, a meat-heavy dietary habit requires large amounts of coenzymes to promote meat digestion. The consumption of these coenzymes may indirectly affect oral health by impacting other bodily functions, thereby increasing the risk of oral frailty. General frailty is a risk factor for oral frailty in MHD patients, consistent with the research results of Tu et al. [12]. Disease burden and dialysis treatment can accelerate the depletion of physiological reserves. Systemic sarcopenia affects swallowing-related muscle groups such as the buccinator, genioglossus, and tongue muscles, leading to weak chewing and swallowing dysfunction in patients [41]. Concurrently, associated fatigue and reduced activity levels decrease patients’ willingness to engage socially and the frequency of oral muscle activity [42], further exacerbating declines in tongue pressure and chewing weakness. This ultimately forms a vicious cycle that mutually exacerbates with systemic frailty [43]. The study results show that MHD patients with depression have a higher prevalence of oral frailty, consistent with the findings of Li et al. [44]. Previous research has indicated that depression is closely associated with dysphagia, oral health status, tooth loss, and the prevalence of xerostomia [45]. It also negatively impacts self-care behaviors related to oral health, accelerating the onset and progression of oral frailty. Future clinical management should, through multidisciplinary collaboration, implement personalized nutritional reinforcement and integrate systematic oral function training, thereby promoting a win-win outcome of synergistic improvement in both systemic condition and oral health.

#### 4.2.3. Family and community networks

At the level of family and community networks, a high level of social support is an independent protective factor against oral frailty in MHD patients, consistent with relevant research findings [46]. In reality, MHD patients often face multiple obstacles in building social support networks. On a macro level, societal cognitive biases may lead to implicit discrimination and a lack of rights advocacy. On an individual level, their employment rate is significantly low [47]. Barriers such as health issues, insufficient support systems, and resource scarcity [48] collectively limit crucial pathways for expanding social connections through occupational means. Therefore, despite MHD patients having a relatively high willingness for social participation, multiple physical and psychological barriers ultimately result in limited actual social engagement [49], making it difficult to maintain regular social life. This decline in social function is closely related to indicators of oral frailty such as perceived decline in chewing ability, tooth loss, and reduced tongue pressure. Oral and maxillofacial disorders, including facial asymmetry, various dentofacial deformities, periodontitis, and tooth loss, not only damage facial esthetics, harmony, and masticatory function but also negatively impact multiple dimensions of an individual’s social function, physical function, and well-being. Conversely, a high level of social support can serve as an informal form of functional exercise by promoting social interaction and communication, helping to delay the decline of both systemic and oral functions, which is crucial for preventing oral frailty.

#### 4.2.4. Working and living conditions

At the level of working and living conditions, the results of this study show that a higher education level and a higher monthly income per family member are significant protective factors. MHD patients with lower education levels had a higher prevalence of oral frailty compared to those with higher education levels, which is consistent with the findings of Tu [12] and Dou et al. [13]. A potential reason is that lower education levels are often associated with limited oral health literacy. This affects patients’ ability to recognize early symptoms of oral diseases, understand complex treatment instructions, and appreciate the importance of long-term oral self-care. Insufficient preventive behaviors and delays in seeking medical care can exacerbate oral problems. Conversely, individuals with higher educational attainment typically possess stronger health awareness, enabling them to proactively seek health knowledge, thereby effectively preventing and controlling the onset and progression of diseases [50]. Research indicates that most MHD patients have high dental care needs [51]. However, the long-term financial burden of the disease itself often impacts their economic status, limiting their access to professional oral healthcare services and consequently accumulating the risk of oral frailty. A higher monthly income provides the economic security needed to access such services, highlighting the socioeconomic gradient in oral health. Therefore, healthcare providers need to pay particular attention to the issue of oral health inequality among low-income groups.

#### 4.2.5. Policy environment

This study did not find statistically significant differences in the impact of different health insurance types on the risk of oral frailty in MHD patients. The reason may stem from the extremely high health insurance coverage rate (97.06%) within the sample, meaning the vast majority of patients already have basic financial protection for medical expenses, thereby weakening the differential effects between policy types. However, high coverage does not equate to adequate oral health protection. Potential underlying reasons include: the current scope and reimbursement ratios of basic health insurance for outpatient oral preventive services (such as regular scaling) and complex dental treatments remain relatively limited. Over 85% of dental expenses are out-of-pocket costs [52], meaning patients still bear most of the financial burden. Economic and time costs remain major barriers; even with health insurance, low-income patients may forgo necessary treatment due to an inability to afford the out-of-pocket portion or difficulty coordinating dialysis and dental appointment times. In regions with higher economic development and more robust public healthcare systems, such as Japan, coverage for dental treatment costs is more comprehensive [53]. Therefore, future policies should focus on deepening the shift from ‘securing basic medical care’ to ‘promoting comprehensive health.’ Exploring the integration of oral health maintenance for MHD patients as part of special disease management, and providing more targeted policy support and healthcare access conveniences, is warranted.

### 4.3. Interrelationships among factors influencing oral frailty in MHD patients

The Bayesian network model constructed in this study not only identified direct influencing factors of oral frailty but also revealed the interrelationships among these factors. (1) Age can indirectly influence the occurrence of oral frailty through general frailty, while depression is indirectly linked to oral frailty *via* poor oral health and general frailty. According to the free radical theory of aging, oxidative damage to macromolecules caused by reactive oxygen species due to advanced age is a pathological basis mediating diseases such as chronic kidney disease [54]. This leads to a decline in the function of bodily tissues and organs, resulting in systemic muscle loss and reduced strength, which affects chewing and swallowing-related muscle groups, thereby weakening oral function. Concurrently, depressive symptoms cause patients to lose interest, experience reduced energy, neglect daily oral hygiene, and decrease the frequency of dental visits [55], accelerating the progression of oral frailty. (2) Education level, monthly income per family member, age, and dietary pattern indirectly affect oral frailty through malnutrition. A higher education level typically corresponds to greater health literacy, while a stable family income provides the material basis for accessing high-quality nutritional resources. Low income limits the ability to obtain quality protein [56]. Furthermore, the preference for soft-textured vegetarian food with increasing age may directly lead to insufficient or imbalanced intake of key nutrients such as dietary fiber and vitamins. Both factors—economic accessibility and food choice—jointly induce or exacerbate malnutrition, which in turn impairs oral health. (3) Eating alone not only mediates the indirect link between social support and oral frailty but is also indirectly associated with oral frailty through dietary pattern and malnutrition. Sharing meals, as an important form of social interaction, can provide social connection and support, promote regular eating behaviors [57], and exercise oral muscles by increasing verbal communication and chewing activity. Conversely, individuals with poor social support, lacking social dining contexts, tend to eat alone. The reduction in social stimulation and swallowing exercise accelerates the decline of oral function, leading to decreased dietary quality, insufficient nutrient intake, and hastened deterioration of oral function.

The results of this study show that when MHD patients present with polypharmacy, malnutrition, eating alone, and general frailty, the probability of developing oral frailty is 92.46%, indicating a high risk. It is recommended that healthcare professionals promptly identify and closely monitor this high-risk group. Forming a multidisciplinary team to collaboratively manage medication and nutritional status, strengthening oral health education and psychological support, encouraging family involvement and social support, and integrating systematic oral function training can establish a closed-loop management system from risk identification to personalized intervention. This approach promotes a win-win outcome of synergistic improvement in both systemic condition and oral health. This also aligns with the HEM, which posits that modifying individual behaviors, optimizing care environments, and integrating clinical pathways can reduce the risk of oral frailty across multiple levels.

### 4.4. Strengths and limitations

The main strength of this study lies in its multi-center design and the systematic exploration of multi-level influencing factors for oral frailty in MHD patients based on the HEM framework, revealing the interrelationships among these factors. The Bayesian network model performed well, visualizing the dependencies between factors and providing a new perspective for targeted intervention. However, this study has certain limitations. As a cross-sectional study with a sample confined to tertiary hospitals, there is a potential for selection bias; thus, caution is needed when extrapolating the conclusions to primary healthcare institutions. Furthermore, although this study explored the causal relationships among variables, the directed arcs in the Bayesian network were generated primarily based on algorithms and correlations, and statistically equivalent alternative structures may still exist. These directed arcs should be interpreted as potential pathways of influence rather than strict causal relationships. Despite low exclusion rate and likely missing completely at random, inability to compare excluded vs. included groups due to incomplete records remains a limitation. Future longitudinal studies are warranted to further validate the causal relationships, thereby providing guidance for the early identification and intervention of oral frailty in MHD patients and enhancing the practical value of the model.

## 5. Conclusion

Oral frailty is relatively prevalent among MHD patients. The study results indicate that polypharmacy, malnutrition, eating alone, and general frailty are directly associated with oral frailty. Age, education level, monthly income per family member, poor oral health, dietary pattern, depression, and social support exert indirect influences on oral frailty. Strengthening the assessment and implementation of oral frailty management through multidisciplinary collaboration is crucial for delaying and potentially reversing the onset and progression of oral frailty. This requires efforts at the levels of individual behavior, care environment, and service integration.

## Data Availability

Data are available from the corresponding author on reasonable request.
